# Circulating microRNAs as novel biomarkers of ALK-positive non-small cell lung cancer and predictors of response to crizotinib therapy

**DOI:** 10.18632/oncotarget.17535

**Published:** 2017-04-30

**Authors:** Liang-Liang Li, Li-Li Qu, Han-Jiang Fu, Xiao-Fei Zheng, Chuan-Hao Tang, Xiao-Yan Li, Jian Chen, Wei-Xia Wang, Shao-Xing Yang, Lin Wang, Guan-Hua Zhao, Pan-Pan Lv, Min Zhang, Yang-Yang Lei, Hai-Feng Qin, Hong Wang, Hong-Jun Gao, Xiao-Qing Liu

**Affiliations:** ^1^ Department of Lung Cancer, Affiliated Hospital of Academy of Military Medical Sciences, Beijing, China; ^2^ Department of Oncology, 309th Hospital of PLA, Beijing, China; ^3^ Department of Biochemistry and Molecular Biology, Beijing Institute of Radiation Medicine, Beijing, China; ^4^ Department of Oncology, Peking University International Hospital, Beijing, China; ^5^ Department of Respiratory, Affiliated Hospital of Aviation Medicine, Beijing, China

**Keywords:** microRNA, plasma, biomarker, non-small cell lung cancer, ALK

## Abstract

Circulating microRNAs are potential diagnostic and predictive biomarkers, but have not been investigated for patients with anaplastic lymphoma kinase (ALK)-positive lung cancer. In this exploratory study, we sought to identify potential plasma biomarkers for ALK-positive non-small cell lung cancer (NSCLC). A microRNA microarray was used to select ALK-related microRNAs in ALK-positive NSCLC (*n* = 3), ALK-negative NSCLC (*n* = 3), and healthy subjects (*n* = 3). Plasma levels of 21 microRNAs were differentially expressed for ALK-positive and ALK-negative NSCLC, including 14 down-regulated and 7 up-regulated microRNAs. We also identified 5s rRNA as the most stable endogenous control gene using geNorm and NormFinder algorithms. Candidate microRNAs in plasma from ALK-positive (*n* = 41) and ALK-negative NSCLC patients (*n* = 32) were quantified using real-time reverse transcriptase quantitative polymerase chain reaction. The expression levels of miR-28-5p, miR-362-5p, and miR-660-5p were all down-regulated in ALK-positive NSCLC, compared with ALK-negative NSCLC. The areas under the receiver operating characteristic curves of miR-28-5p, miR-362-5p, miR-660-5p, and 3-microRNAs panel were 0.873, 0.673, 0.760, and 0.876, respectively. The positive predictive values of miR-28-5p, miR-362-5p, and miR-660-5p were 96.43%, 80.77%, and 83.87%, respectively. Increased plasma levels of miR-660-5p after crizotinib treatment predicted good tumor response (*p* = 0.012). The pre-crizotinib levels of miR-362-5p were significantly associated with progression-free survival (*p* = 0.015). Thus, in this preliminary investigation, we identified a potential panel of 3 microRNAs for distinguishing between patients with ALK-positive and ALK-negative NSCLC. We also identified miR-660-5p and miR-362-5p as potential predictors for response to crizotinib treatment.

## INTRODUCTION

In 2007, investigators in Japan identified anaplastic lymphoma kinase (ALK) as a novel potential target in NSCLC. ALK rearrangements occur in 3% to 7% of patients with NSCLC. Most ALK-positive lung cancer patients are characterized by younger ages, no history of smoking or a history of light smoking, and adenocarcinoma histology [1, 2]. Patients who have advanced ALK-positive NSCLC are highly responsive to the ALK inhibitor crizotinib, with an objective response rate of approximately 60% and a median progression-free survival of 8 to 10 months [3, 4]. The current standard method for detecting ALK-positivity in NSCLC is fluorescence *in situ* hybridization (FISH) [5]. However, it is sometimes difficult to collect tumor tissues from patients with advanced-stage NSCLC. It has been shown that circulating biomarkers are present in plasma and other body fluids, and may have potential as novel, non-invasive biomarkers [6–8]. Recent findings indicate that circulating microRNAs are useful as non-invasive biomarkers for different tumor types, including lung cancer [9–16].

MicroRNAs are a class of evolutionarily conserved, small (18 to 24 nucleotides), non-coding RNA molecules that play key roles in the regulation of gene expression by base pairing to the complementary sites in their target mRNAs [17–19]. Increasing evidence has shown that microRNAs have more important roles in tumor development than had previously been anticipated; specifically, they are believed to act as tumor suppressors or oncogenes in a wide range of human cancers [20–22].

In the past decade, it was found that microRNAs are stably expressed in human blood, and that circulating microRNAs may serve as disease fingerprints and novel molecular biomarkers for cancer [23, 24]. Blood-based biomarkers have several significant advantages: they can be obtained easily and in a minimally invasive manner, they can be measured repeatedly, and they can be compared longitudinally. However, circulating microRNAs in plasma have not been systematically and extensively studied in ALK-positive lung cancer. Therefore, in this exploratory study, we sought to identify a plasma microRNA panel that could effectively separate ALK-positive NSCLC patients from ALK-negative individuals. Furthermore, we sought to explore the potential of the microRNA panel as a non-invasive predictive biomarker for response to crizotinib.

## RESULTS

### Clinical characteristics

**Figure 1:**
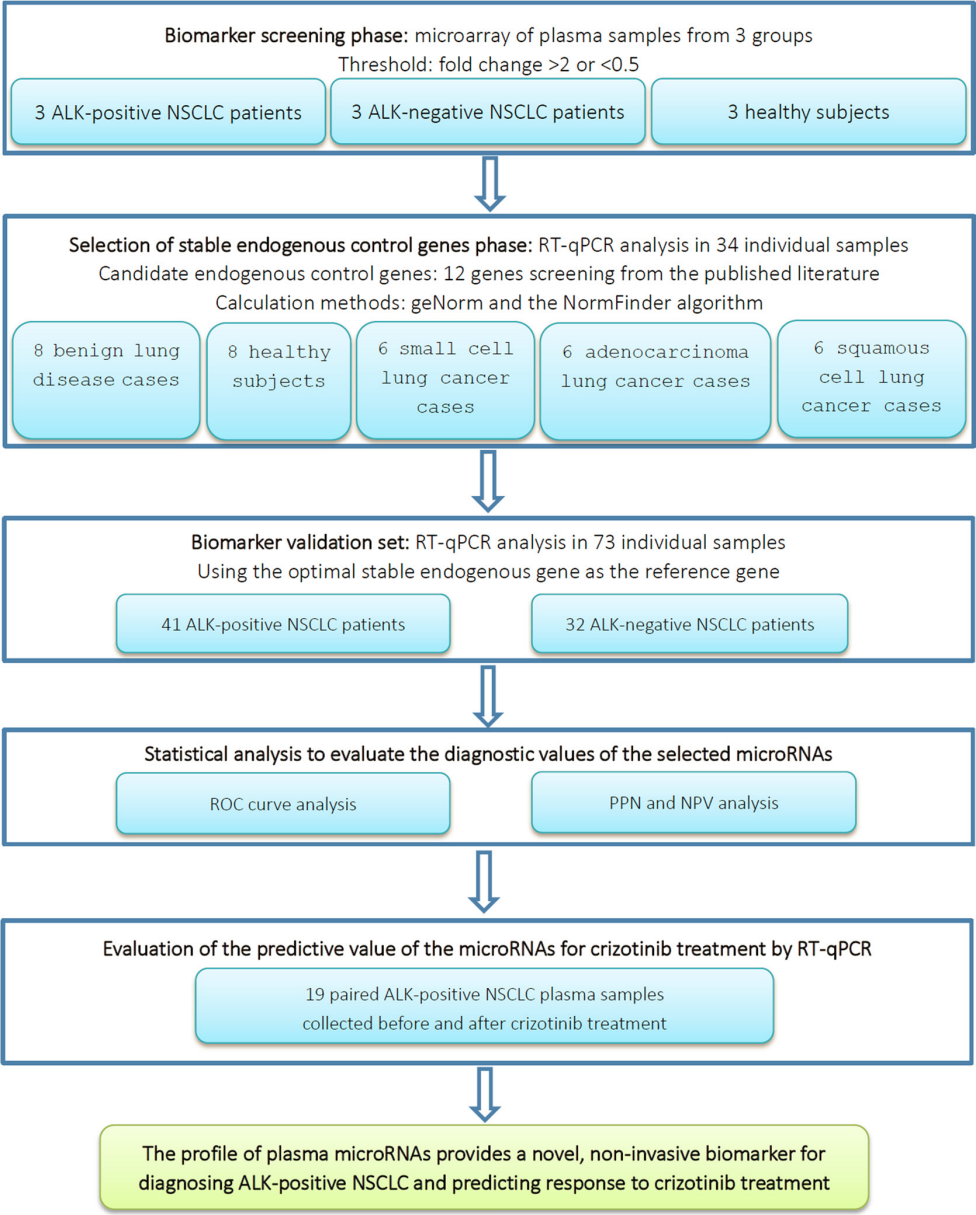
Overall study design and numbers of included patients ALK, anaplastic lymphoma kinase; NPV, negative predictive value; NSCLC, non-small cell lung cancer; PPV, positive predictive value; ROC, receiver operating characteristic curve; RT-qPCR, real-time quantitative polymerase chain reaction.

A total of 73 participants, including 41 patients with ALK-positive lung cancer and 32 patients with ALK-negative lung cancer, were enrolled in the validation phase of the present study. The overall study design is shown in Figure 1. The clinical features of the participants are listed in Table 1. There were no significant differences in gender, clinical stage, or pathology between the patients with ALK-positive and ALK-negative disease. However, there were significant differences in clinical characteristics such as age and smoking history. Compared with ALK-negative NSCLC patients, ALK-positive NSCLC patients tended to be younger and have little or no smoking histories. The median ages of patients with ALK-positive and ALK-negative NSCLC were 50 years (range, 28–69) and 61.4 yeas (range, 34–84), respectively. The proportion of never-smokers in the ALK-positive group was significantly higher than that in the ALK-negative group (68.3% vs. 43.8%).

**Table 1:** Patient characteristics at the time of primary diagnosis

Clinical variable	ALK-positive NSCLC (*n* = 41)	ALK-negative NSCLC (*n* = 32)	*p* value
Age			0.002
Median (range)	50.0 (28–69)	61.4 (34–84)	
Gender			0.526
Male	20 (48.8%)	18 (56.3%)	
Female	21 (51.2%)	14 (43.8%)	
Stage			0.194
III	4 (9.8%)	7 (21.9%)	
IV	37 (90.2%)	25 (78.1%)	
Smoking history			0.043
Never smokers	28 (68.3%)	14 (43.8%)	
Light smokers (smoking pack-years < 30)	9 (22.0%)	8 (25.0%)	
Heavy smokers (smoking pack-years ≥ 30)	4 (9.8%)	10 (31.3%)	
Histologic subtype			0.189
Adenocarcinoma	41 (100%)	30 (93.8%)	
Squamous carcinoma	0	0	
Others	0	2 (6.3%)	
Differentiation			0.587
G1	0	0	
G2	3 (7.3%)	3 (9.4%)	
G3	19 (46.3%)	11 (34.4%)	
Gx	19 (46.3%)	18 (56.3%)	

ALK, anaplastic lymphoma kinase; NSCLC, non-small cell lung cancer. G1: low grade or well differentiated cancer cells, which still closely resemble normal cells; G2: intermediate/moderate grade or moderately differentiated cancer cells, which do not look like normal cells; G3: high grade or poorly differentiated cancer cells, which do not look like normal cells at all; Gx: grade unknown.

### Selection of differential microRNAs by microarray

**Figure 2:**
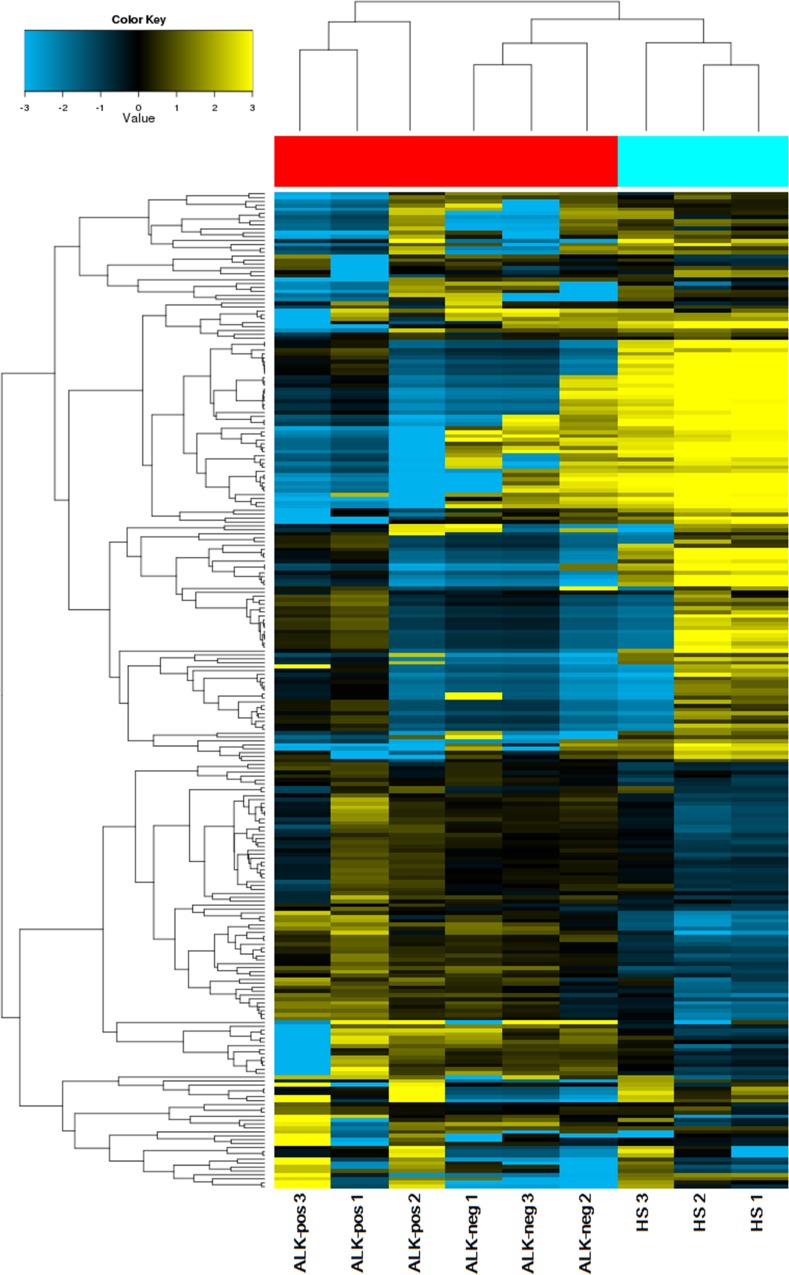
Cluster analysis of microRNA expression profiles in plasma from ALK-positive NSCLC, ALK-negative NSCLC, and HS cohorts

In our investigation, circulating microRNAs from plasma samples of patients with ALK-positive NSCLC (*n* = 3), patients with ALK-negative NSCLC (*n* = 3), and healthy subjects (*n* = 3) were analyzed by microarray (Human miRNA Microarray, G4872A; Agilent Technologies, Santa Clara, CA, USA; Gene Expression Omnibus accession no. GSE94536) and real-time reverse transcriptase quantitative polymerase chain reaction (RT-qPCR) technologies. Among the 2,568 microRNAs assessed with the microarray technology, 438 microRNAs were detected in at least 1 out of the 9 samples analyzed. It is worth noting that the total numbers of circulating microRNAs differed in the 3 cohorts. Compared with the cohorts of patients with NSCLC, more microRNAs were detected in the plasma of healthy subjects. Specifically, 409 microRNAs were detected in healthy subjects, whereas 116 were detected in patients with ALK-positive NSCLC and 209 were detected in patients with ALK-negative NSCLC (Figure 2).

We identified 21 microRNAs that were differentially expressed in plasma between patients with ALK-positive NSCLC and those with ALK-negative NSCLC. Compared with the ALK-negative group, plasma from the ALK-positive group had 7 up-regulated microRNAs and 14 down-regulated microRNAs (fold change > 2 or < 0.5) (Table 2).

**Table 2:** MicroRNAs that were found to be dysregulated between patients with ALK-positive and ALK-negative non-small cell lung cancer, based on microarray analysis

*N*	Systematic name	ALK-pos vs. ALK-neg		
		Regulation	FC	*p* value
1	miR-16-5p	down	9.44	0.246
2	miR-17-5p	down	12.33	0.009
3	miR-19a-3p	down	27.72	0.002
4	miR-20a-5p	down	31.06	0.002
5	miR-22-3p	down	9.42	0.123
6	miR-24-3p	down	32.86	0.008
7	miR-25-3p	down	36.01	0.002
8	miR-28-5p	down	14.87	0.005
9	miR-96-5p	down	10.55	0.020
10	miR-107	down	30.23	0.002
11	miR-340-5p	down	2.89	0.060
12	miR-483-3p	up	30.196	0.038
13	miR-579-5p	up	2.50	0.034
14	miR-619-5p	up	10.93	0.021
15	miR-660-5p	down	34.51	0.006
16	miR-3195	down	8.45	0.038
17	miR-4306	down	42.59	0.001
18	miR-6751-3p	up	32.86	0.046
19	miR-6779-3p	up	14.34	0.021
20	miR-6797-3p	up	17.38	0.030
21	miR-6858-5p	up	30.03	0.037

ALK, anaplastic lymphoma kinase; ALK-pos, ALK-positive; ALK-neg, ALK-negative; FC, fold change.

### Identification of the endogenous control genes

**Figure 3:**
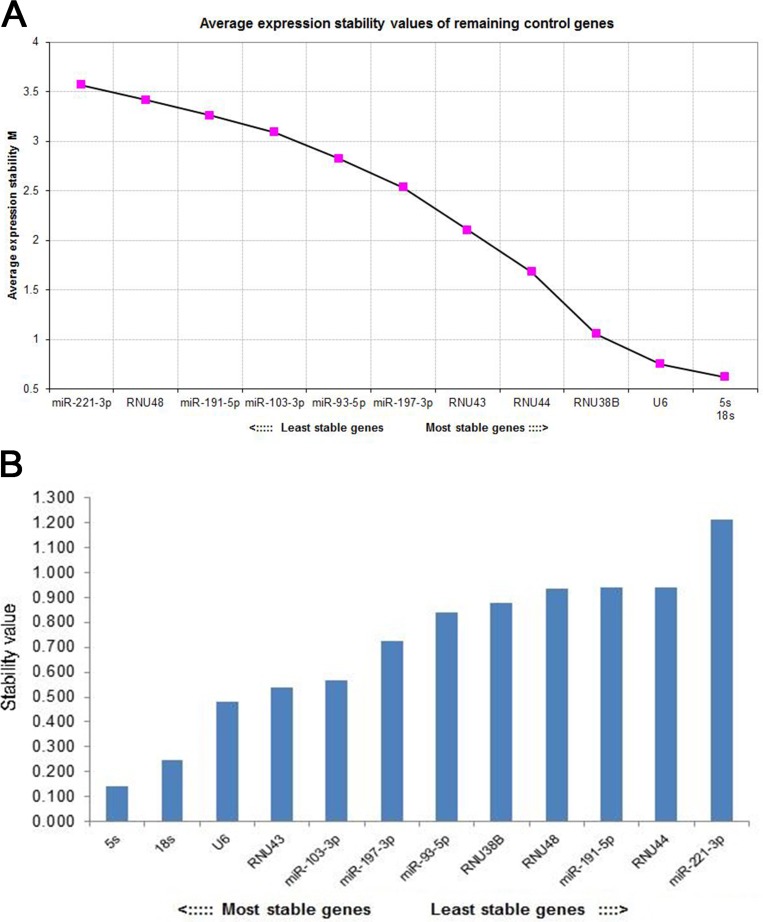
The stability of candidate endogenous genes was ranked using geNorm and the NormFinder algorithm (**A**) Selection of the most stable reference genes from a panel of 12 genes using geNorm. The geNorm program calculated the average expression stability value (M) for each gene. Starting from the least stable genes at the left, the genes were ranked according to increasing expression stability, ending with the 2 most stable genes at the right. Candidates 5 s and 18 s were the 2 most stable genes. (**B**) Identification of the most stable reference gene or gene combination using NormFinder. The NormFinder algorithm ranks the set of candidate normalization genes according to their expression stability in different groups. When using this algorithm, greater gene stability is indicated by a lower stability value for the individual gene. Candidate 5 s was the most stable gene.

As shown in Figure 3, the geNorm and NormFinder algorithms were used to rank the candidate reference genes according to their expression stability. Based on calculations performed with geNorm, 5s rRNA had the most stable expression level and thus was selected as the endogenous control gene. NormFinder confirmed the results that had been obtained by geNorm, showing that 5s rRNA was the most stable reference gene. Several other genes that are commonly used as reference genes for microRNA RT-qPCR experiments ranked behind 5s rRNA, suggesting that they should not be considered suitable reference genes for this study. These genes included 18s rRNA, RNU6B, RNU38B, RNU44, RNU43, RNU48, miR-221-3p, miR-93-5p, miR-191-5p, miR-103-3p, and miR-197-3p.

### Selection of candidate plasma microRNAs for ALK-positive NSCLC

According to microarray analysis, the levels of 21 microRNAs were differentially expressed between patients with ALK-positive and ALK-negative NSCLC. Of these differentially expressed microRNAs, 14 were down-regulated and 7 were up-regulated in ALK-positive NSCLC, as compared with ALK-negative NSCLC (Table 2). To increase the likelihood of identifying useful plasma biomarkers, we focused on the microRNAs that showed the highest degrees of dysregulation in the ALK-positive NSCLC samples (fold-change in comparison to ALK-negative NSCLC) and that were among the most abundant in terms of absolute expression levels. In addition, we searched the published literature for microarray data on ALK-positive and ALK-negative NSCLC tissue [25]. Inconsistent expression levels between plasma samples and tissue samples were observed. Comparing our plasma microarray data with the tissue microarray data based on the published literature, 2 microRNAs (miR-362-5p and miR-579-5p) with high expression differences in tissue microarray findings were added.

Based on these criteria, we selected 13 microRNAs for further analysis in plasma by RT-qPCR: miR-16-5p, miR-17-5p, miR-19a-3p, miR-20a-5p, miR-22-3p, miR-362-5p, miR-24-3p, miR-28-5p, miR-96-5p, miR-107, miR-340-5p, miR-579-5p, and miR-660-5p. The primers are listed in Table 3.

**Table 3:** MicroRNA primers

MicroRNA	Primer sequence (5′ to 3′)
5s rRNA Forward	TCTGATCTCGGAAGCTAAGCA
5s rRNA Reverse	CCTACAGCACCCGGTATTCC
miR-16-5p	TAGCAGCACGTAAATATTGGCG
miR-17-5p	CAAAGTGCTTACAGTGCAGGTAG
miR-19a-3p	TGTGCAAATCTATGCAAAACTGA
miR-20a-5p	TAAAGTGCTTATAGTGCAGGTAG
miR-22-3p	AAGCTGCCAGTTGAAGAACTGT
miR-24-3p	TGGCTCAGTTCAGCAGGAACAG
miR-28-5p	AAGGAGCTCACAGTCTATTGAG
miR-96-5p	TTTGGCACTAGCACATTTTTGCT
miR-107	AGCAGCATTGTACAGGGCTATCA
miR-340-5p	TTATAAAGCAATGAGACTGATT
miR-362-5p	AATCCTTGGAACCTAGGTGTGAGT
miR-579-5p	TCGCGGTTTGTGCCAGATGACG
miR-660-5p	TACCCATTGCATATCGGAGTTG

### Validation of candidate microRNAs by RT-qPCR

**Figure 4:**
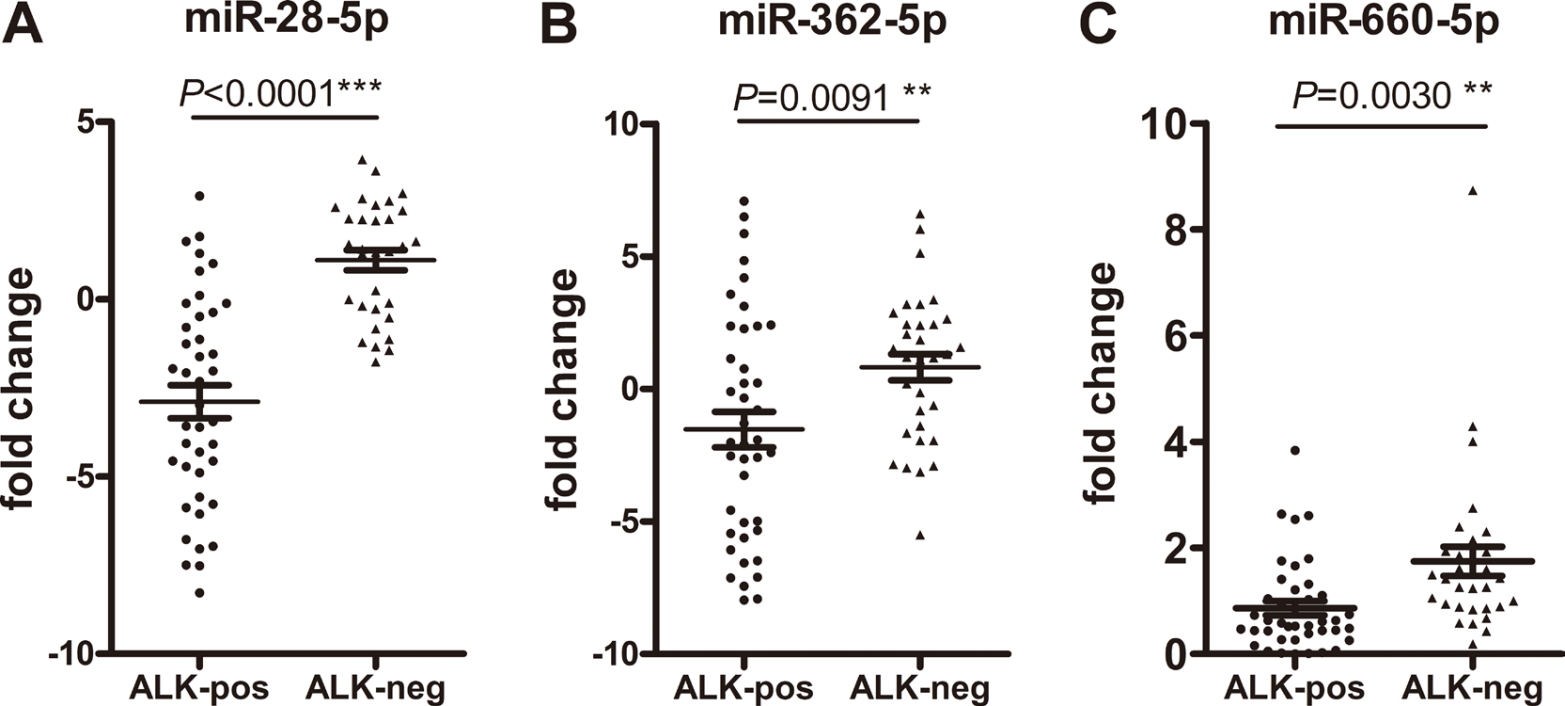
Plasma levels of miR-28-5p, miR-362-5p, and miR-660-5p are reduced in patients with anaplastic lymphoma kinase (ALK)-positive non-small cell lung cancer The microRNA expression levels in the ALK-positive and ALK-negative non-small cell lung cancer cohorts (x-axis) are shown as 2^−ΔΔCt^ values (y-axis), as calculated from real-time quantitative polymerase chain reaction. Statistically significant *p* values are indicated as follows: *p < 0.05; **p < 0.01; ***p < 0.001.

The 13 microRNAs selected from the microarray results were first confirmed in the validation cohort, which included 41 ALK-positive NSCLC cases and 32 ALK-negative NSCLC cases. Only the microRNAs with a mean fold change > 2 or < 0.5 and a *p value* < 0.5 were selected for further validation. Three of the 13 microRNAs were markedly dysregulated in plasma according to ALK status. These 3 microRNAs, miR-28-5p, miR-362-5p, and miR-660-5p, were all down-regulated (Figure 4).

**Figure 5:**
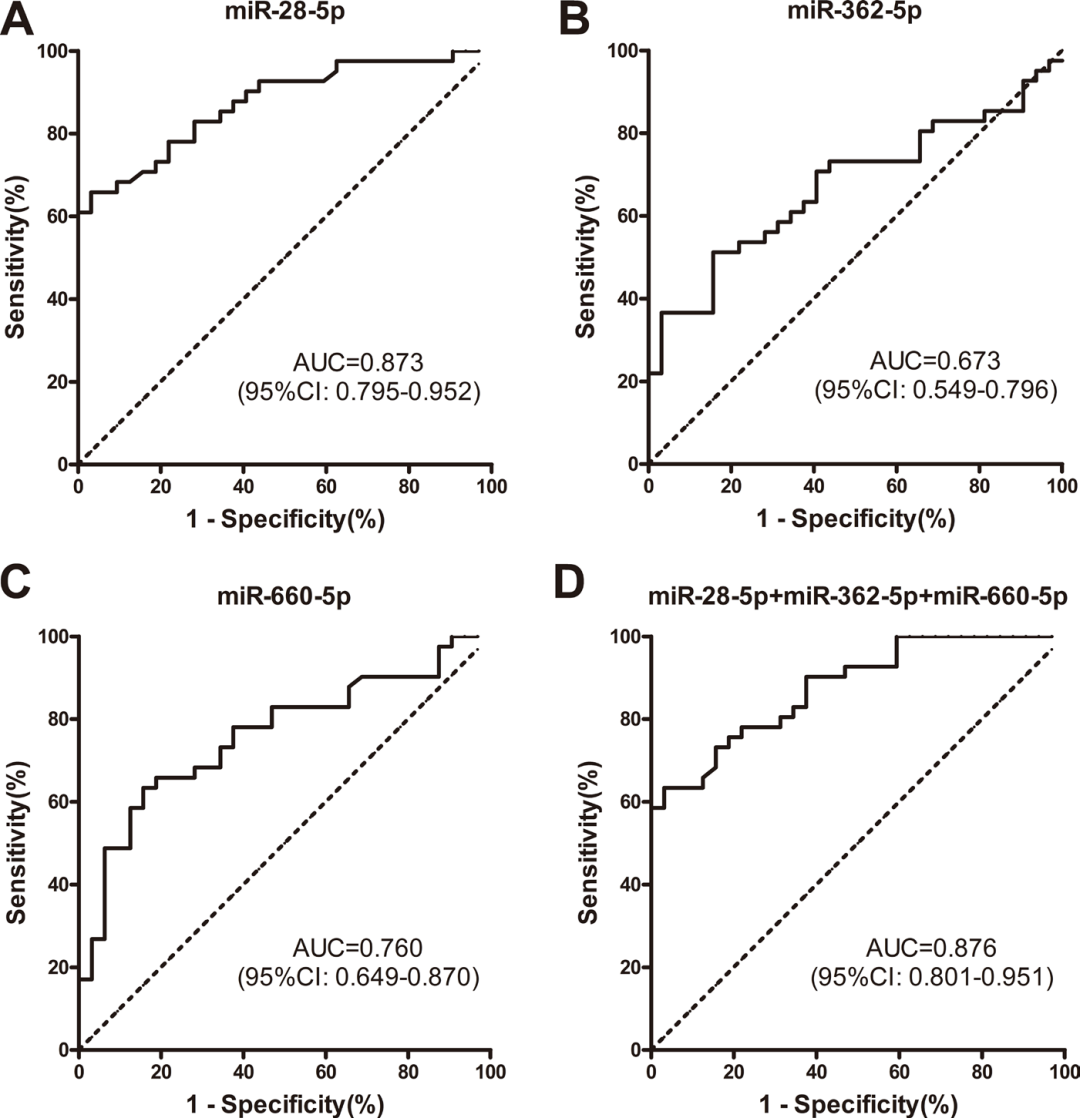
ROC curve analyses demonstrated that plasma levels of miR-28-5p, miR-362-5p, and miR-660-5p differed between patients with ALK-positive and ALK-negative NSCLC (**A**–**C**) ROC curves showing that plasma levels of miR-28-5p, miR-362-5p, and miR-660-5p differ between patients with ALK-positive and ALK-negative NSCLC. (**D**) Combination ROC curve analyses of the 3 microRNAs as a means of distinguishing between patients with ALK-positive and ALK-negative NSCLC. ALK, anaplastic lymphoma kinase; AUC, area under the curve; NSCLC, non-small cell lung cancer; ROC, receiver operating characteristic curve.

Subsequently, we conducted receiver operating characteristic (ROC) curve analyses on each of the 3 individual plasma microRNAs, to assess their diagnosis values for distinguishing between patients with ALK-positive and ALK-negative NSCLC. To determine whether the plasma levels of miR-28-5p, miR-362-5p, and miR-660-5p had the abilities to discriminate between the ALK-positive and ALK-negative groups, ROC curves were used to estimate their diagnostic sensitivities and specificities (Figure 5; Table 4).

**Table 4:** Measures of diagnostic performance for distinguishing patients with ALK-positive and ALK-negative non-small cell lung cancer

Variables	miR-28-5p	miR-362-5p	miR-660-5p
Cut-off	−1.493	−1.984	0.7902
Sensitivity	65.85%	51.22%	63.41%
Specificity	96.88%	84.38%	84.38%
PPV	96.43%	80.77%	83.87%
NPV	68.89%	57.45%	64.29%

ALK, anaplastic lymphoma kinase; PPV, positive predictive value; NPV, negative predictive value.

Based on an optimal cut-off value (−1.493) that was chosen using Youden's Index, the sensitivity and specificity of miR-28-5p were 65.85% and 96.88% for distinguishing patients with ALK-positive NSCLC from those with ALK-negative NSCLC (area under curve [AUC] = 0.873, 95% CI, 0.795–0.952). With a threshold of −1.984 (AUC = 0.673, 95% CI, 0.549–0.796), and the sensitivity and specificity of miR-362-5p were 51.22% and 84.38%. With a threshold of 0.7902, the sensitivity and specificity of miR-660-5p were 63.41% and 84.38% (AUC = 0.760, 95% CI, 0.649-0.870). Furthermore, combining the 3 microRNAs increased the AUC (0.876, 95% CI, 0.801–0.951) and specificity (96.88%), but decreased the sensitivity (63.41%) for distinguishing between the ALK-positive and ALK-negative groups.

To clarify the diagnostic capability of the combination of the 3 microRNAs, we also estimated the positive predictive value (PPV) and negative predictive value (NPV) for distinguishing ALK-positive patients from ALK-negative patients. As shown in Table 4, the PPV for the 3 microRNAs was higher than 80%, which indicated that a non-small cell lung cancer patient with low plasma levels of any 1 of the 3 microRNAs was more likely to have ALK-positive disease compared with a patient with high plasma levels of the 3 microRNAs. In addition, the NPV of the 3 microRNAs was higher than 64%. Overall, the results indicate that the plasma levels of the 3 microRNAs have high diagnostic value.

### The changes in plasma levels of miR-660-5p after crizotinib treatment predict tumor response

**Figure 6:**
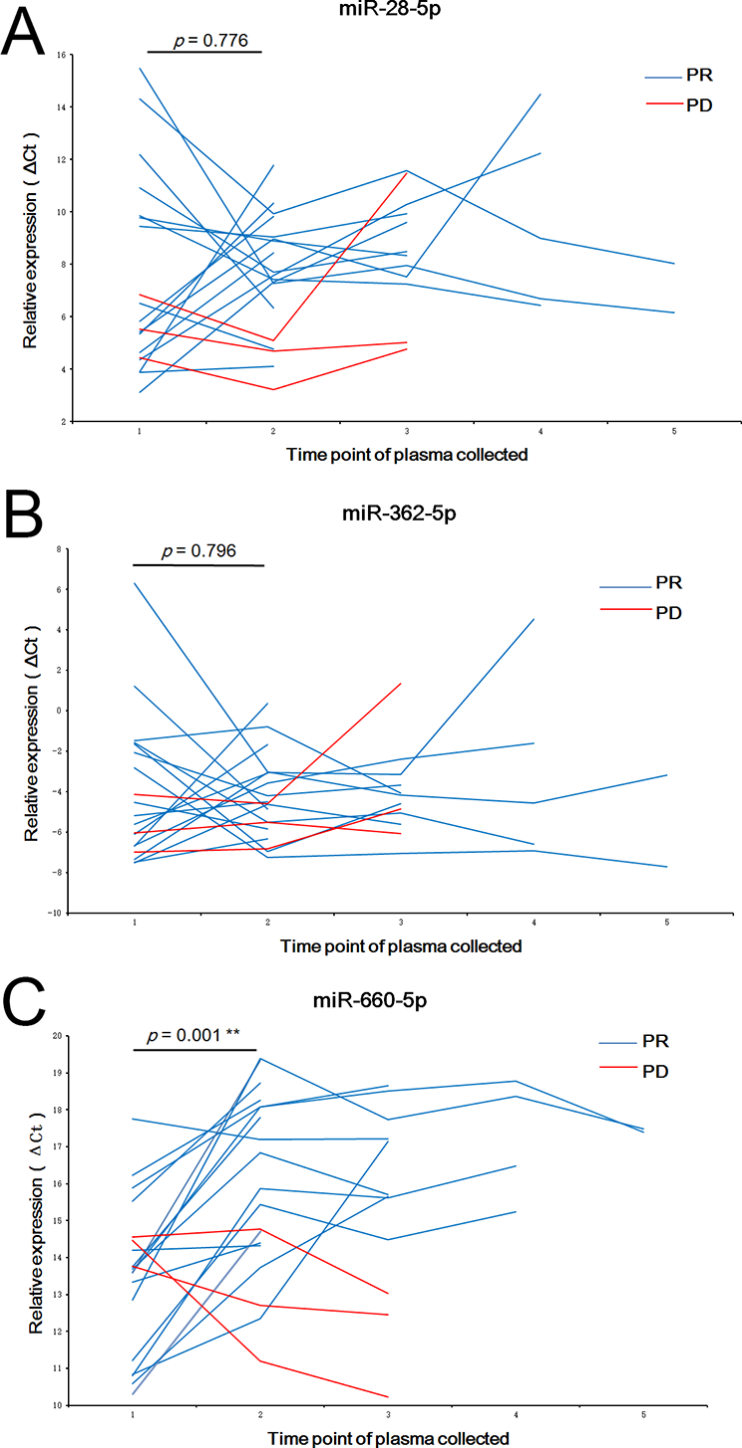
The changes of plasma levels of miR-28-5p, miR-362-5p, and miR-660-5p before and after treatment with crizotinib The y-axes show the fold change of miR-28-5p (**A**) miR-362-5p (**B**) and miR-660-5p (**C**) which were normalized to 5s rRNA.

To determine whether the plasma levels of miR-28-5p, miR-362-5p, and miR-660-5p had predictive value for response to crizotinib therapy, the plasma levels of the 3 microRNAs in 19 patients were compared before and after crizotinib treatment using RT-qPCR. Fifty-seven plasma samples were collected from the 19 patients at time points before and after treatment with crizotinib. The blood samples were collected for the first time 2 weeks (±2 days) before crizotinib and then every 2 months (±1 week) thereafter. At the same times, chest computed tomography was performed to evaluate response to therapy. Of the 19 patients, 16 had a partial response and 3 had progressive disease. Pre- and post-treatment microRNA levels are shown in Figure 6, and were analyzed using the Wilcoxon matched pairs signed rank sum test. The plasma levels of miR-660-5p increased after crizotinib treatment in 15 (93.75%) of the 16 patients with partial response (*p* = 0.001). In contrast, a similar trend was not observed in the remainder of the patients, who had progressive disease. The plasma level changes of miR-28-5p and miR-362-5p after crizotinib treatment were not statistically significant (*p* = 0.776 and *p* = 0.796, respectively). Overall, our investigation of the 3 microRNAs showed that changes in the plasma level of miR-660-5p before and after crizotinib treatment had value as a predictor of good tumor response.

### The relationship between the initial plasma levels of microRNAs and the progression-free survival of patients treated with crizotinib

**Figure 7:**
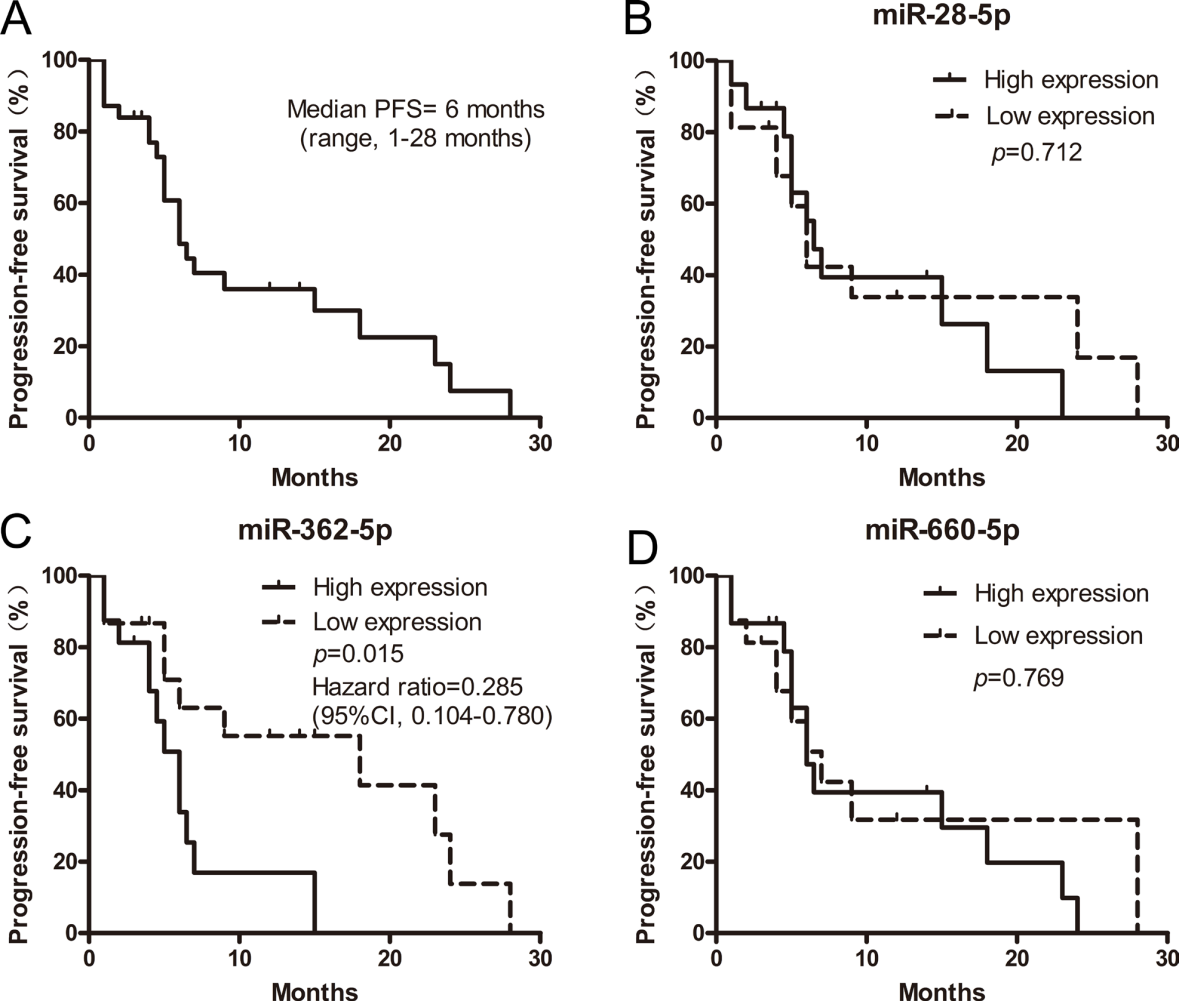
The relationship between the initial expression levels of plasma microRNAs and the progression-free survival (PFS) of ALK-positive patients receiving crizotinib (*n* = 31) (**A**) PFS of ALK-positive patients who received crizotinib therapy. (**B**) PFS of ALK-positive patients, stratified according to the expression levels of miR-28-5p. (**C**) PFS of ALK-positive patients, stratified according to the expression levels of miR-362-5p. (**D**) PFS of ALK-positive patients, stratified according to the expression levels of miR-660-5p. All survival rates were estimated using the Kaplan-Meier method.

Of 41 patients with ALK-positive NSCLC, 31 received crizotinib therapy. We performed Kaplan-Meier analyses of progression-free survival (PFS) for clinicopathological factors and the initial (pre-crizotinib) plasma levels of miR-28-5p, miR-362-5p, and miR-660-5p. Because 30 of the 31 patients had a performance status of 1, and only 1 patient had a performance status of 0, performance status was excluded from the Kaplan-Meier analyses. The expression levels of the 3 microRNAs in plasma were first stratified by their median values. Next, the PFS of the patients with high microRNA expression levels (≥ median) was compared with the PFS of patients with low microRNA expression levels (< median). As shown in Figure 7, the median PFS of the 31 patients was 6 months (range, 1–28). The initial expression levels of miR-28-5p and miR-660-5p had no significant association with PFS (*p* = 0.712 and *p* = 0.7686, respectively). However, the expression levels of miR-362-5p were significantly associated with PFS (*p* = 0.015), showing the predictive value. For patients with high and low expression levels of miR-362-5p, the median PFS times were 18 and 6 months, respectively (hazard ratio, 0.285; 95% confidence interval, 0.104–0.780), respectively. As shown in Table 5, smoking history was significantly associated with PFS (hazard ratio, 0.01; 95% confidence interval, 0.00–0.06; *p* = 0.001). These results suggest that miR-362-5p and smoking history were both important predictive factors for ALK-positive NSCLC patients receiving crizotinib therapy. Next, a multivariate analysis was performed, including all of the factors that showed significant associations with PFS in the Kaplan-Meier analyses. Clinical stage was also included, considering its correlation with survival. In the multivariate analysis, lower expression of miR-362-5p was significantly associated with longer PFS. However, smoking history and stage were not independent prognostic factors for PFS (Table 6).

**Table 5:** Kaplan-Meier analysis of factors associated with progression-free survival

Clinical variables	Hazard ratio (95% CI)	*p* value
Gender (male vs. female)	1.88 (0.75–4.70)	0.177
Age (≥ 60 years vs. < 60 years)	0.71 (0.27–1.86)	0.485
Stage (IIIB vs. IV)	0.58 (0.17–1.96)	0.379
Smoking history (pack-years < 30 vs. pack-years ≥ 30)	0.01 (0.00–0.06)	0.001
Differentiation (G2 vs. G3)	1.33 (0.12–14.28)	0.813
miR-28-5p (low-expression vs. high-expression )	0.84 (0.34–2.10)	0.712
miR-362-5p (low-expression vs. high-expression )	0.29 (0.10–0.78)	0.015
miR-660-5p (low-expression vs. high-expression )	0.87 (0.36–2.14)	0.769

CI, confidence interval; G2, intermediate/moderate grade; G3, high grade.

Progression-free survival was analyzed using the Kaplan-Meier method, and Hazard ratios were estimated using the Logrank test.

**Table 6:** Multivariate analysis of factors associated with progression-free survival

Clinical variables	Progression-free survival	
	Hazard ratio (95% CI)	*p* value
Stage (IV vs. IIIB)	1.85 (0.42–8.18)	0.419
Smoking history (pack-years ≥ 30 vs. pack-years < 30)	5.96 (0.97–36.68)	0.054
miR-362-5p (low-expression vs. high-expression)	0.32 (0.12–0.88)	0.027

CI, confidence interval.

Hazard ratios were estimated using the multivariate Cox proportional regression hazards model.

## DISCUSSION

In this study, we evaluated the potential of circulating microRNAs as novel biomarkers of ALK status and response to crizotinib in patients with NSCLC.

To our knowledge, our report is the first to describe a prospective analysis of circulating microRNAs as diagnostic or prognostic biomarkers for ALK-positive lung cancer that is treated with crizotinib. We identified a new panel of 3 microRNAs (miR-28-5p, miR-362-5p, and miR-660-5p) that can distinguish ALK-positive NSCLC from ALK-negative NSCLC with high specificity and sensitivity. The plasma levels of the 3 microRNAs were all down-regulated in ALK-positive NSCLC patients, as compared with ALK-negative NSCLC patients. For each of the 3 microRNAs, the PPV exceeded 80%. Furthermore, increased plasma levels of miR-660-5p after treatment with crizotinib were correlated with good tumor response in patients who had ALK-positive NSCLC. Our results suggest that this panel of 3 microRNAs has considerable potential as an auxiliary diagnostic, and that dynamic monitoring of microRNA biomarkers could be useful for ALK-positive NSCLC patients receiving crizotinib treatment.

In previous studies of NSCLC, several different reference control genes were reported, including small RNAs, endogenous microRNAs, and external non-human microRNAs. At present, there is no known, optimal, stable endogenous control gene in the plasma of patients with NSCLC. In the present study, 12 candidate reference genes were analyzed using the geNorm and NormFinder algorithms. These algorithms confirmed that 5s rRNA was the most stable endogenous control gene in the cohorts of patients with lung cancer, patients with benign lung diseases, and health subjects. The use of this appropriate reference for the normalization of microRNAs in plasma improves the sensitivity and reproducibility of the results and helps to ensure reliable biomarker discovery.

To date, no report has described correlations between miR-28-5p and lung cancer or the ALK pathway. Research on expression levels of miR-28-5p in other cancers has shown inconsistent results [26–34]. Most of these prior reports have demonstrated that miR-28-5p is down-regulated in cancer tissue or blood. However, another report noted that miR-28 is stable in esophageal cancer tissue, and can be used as internal reference gene [26]. In our study, plasma levels of miR-28-5p were down-regulated in ALK-positive NSCLC patients, as compared with ALK-negative NSCLC patients.

Although we performed a search of the literature, we did not find any studies that investigated the associations between miR-362-5p and ALK-positivity in patients with lung cancer. However, in studies of neuroblastoma and gestational diabetes mellitus, miR-362-5p was found to be involved in the PI3K pathway [35, 36]. As is commonly known, PI3K is a downstream signaling pathway of the ALK pathway. Therefore, miR-362-5p may be associated with ALK status through signal transducer and activator of transcription (STAT).

We know little about the role of miR-660-5p in tumors. Some studies have shown that miR-660 is a prognostic marker in breast cancer [37], and is also down-regulated in Hodgkin's lymphoma [38]. In the present study, miR-660-5p was decreased in patients with ALK-positive NSCLC. MiR-660-5p can not only be used as a diagnostic marker for ALK-positive lung cancer, but is also associated with the efficacy of crizotinib.

There are several limitations to this study. First, the participant samples were collected from a single lung cancer center. Further external validation of our results would require investigating samples from multiple centers. Second, the number of participants was relatively small. Future work will require a larger number of samples to ensure the reliability of the 3-microRNA panel. Third, the present study failed to investigate and compare the expressions of identified microRNAs in plasma and tissue from the same individuals. Future studies will require pairs of plasma and tissue sample from the same individuals, to verify whether expression levels of microRNAs in plasma and tissue are consistent.

In conclusion, in this preliminary and exploratory study, we established a plasma microRNA panel that may serve as a novel, non-invasive biomarker for the diagnosis of ALK-positive NSCLC, and may provide considerable value as a predictor of tumor response to treatment with crizotinib.

## MATERIALS AND METHODS

### Participants and study design

From December 2014 to August 2016, we enrolled a total of 116 participants from the Department of Lung Cancer and the Physical Examination Center at the Affiliated Hospital of Academy of Military Medical Science, including 91 patients with NSCLC, 6 patients with small cell lung cancer, 8 patients with benign lung disease, and 11 healthy subjects. Our study had a single-center, prospective, and clinical design. In the study cohort, the participants who had ALK-positive or ALK-negative NSCLC were consecutively enrolled, whereas the other participants were randomly enrolled. The inclusion criteria for ALK-positive NSCLC were (1) histological confirmed advanced NSCLC, (2) age ≥ 18 years, (3) expected survival more than 3 months, (4) ALK rearrangement measured by FISH before crizotinib, and (5) no history of ALK inhibitor therapy. Patients who had multiple tumors were excluded. All lung cancer patients were diagnosed by histopathological analysis. The clinical stage was assessed by 2 independent professional oncologists according to the American Joint Committee on Cancer (AJCC, 7th edition)/Union for International Cancer Control (UICC) tumor, node, metastasis (TNM) staging system. Each lung cancer patient had a performance status within 0 to 1, as evaluated according to the Eastern Cooperative Oncology Group (ECOG) criteria. Tumor assessments were performed at baseline and every 2 months during treatment until progression. Objective tumor response was evaluated following the Response Evaluation Criteria in Solid Tumors (RECIST). The healthy subjects were age- and gender-matched individuals who had no history of cancer and were in a condition of good health, based on the findings of physical examinations. In our study, patients with benign lung disease refer to patients with pneumonia who had been diagnosed by computed tomography scanning and bacteriological examination. This study was performed with the approval of the Affiliated Hospital of Academy of Military Medical Science Ethics Committee. Informed written consent was obtained from all patients/subjects.

To identify microRNAs that showed differential expression associated with ALK status, we first used microarray analysis in a discovery cohort consisting of 9 individuals: 3 ALK-positive NSCLC patients, 3 ALK-negative NSCLC patients, and 3 healthy subjects. Subsequently, RT-qPCR assays were used to identify the most stable circulating endogenous gene in 34 individuals (8 healthy subjects, 8 patients with benign lung disease, 6 patients with small cell lung cancer, 6 patients with squamous cell lung cancer, and 6 patients with adenocarcinoma lung cancer). Finally, we evaluated the plasma microRNAs by RT-qPCR assays from the validation cohort, which included 41 patients with ALK-positive NSCLC patients and 32 patients with ALK-negative NSCLC.

In addition, paired plasma samples were collected from 19 patients in the validation cohort before and after treatment with crizotinib. The plasma from these patients was analyzed using RT-qPCR to identify whether the microRNAs biomarkers were predictive of response to crizotinib. In the validation cohort, 41 patients were confirmed as having ALK-positive tumors at the initial diagnosis by FISH and 32 were ALK-negative. Of the 41 ALK-positive patients, 31 patients received treatment with crizotinib at an initial dose of 250 mg twice daily, with appropriate dosing modification as needed. Of the 31 patients receiving crizotinib treatment, 19 had plasma samples taken at both the initial diagnosis and 2 months after treatment with crizotinib. The remaining 12 patients had plasma samples taken at the initial diagnosis only. The overall study design is shown in Figure 1. The characteristics of the 73 patients are summarized in Table 1.

### Plasma sample collection and RNA isolation

For plasma collection, venous blood (5 mL) was collected and placed at room temperature for 1 hour, and then centrifuged at 1600 *g* for 10 min at 4°C. The plasma was then divided into small aliquots and frozen at −80°C until use. RNA was isolated from plasma using miRNeasy Mini Kit (Qiagen, Valencia, CA) following the manufacturer's protocol. In short, 200 μL of EDTA-plasma were mixed with 1 mL of Qiazol Lysis Reagent, incubated for 5 min at room temperature, and subsequently mixed with 200 μL of chloroform. The organic and aqueous phase was separated by centrifugation at 12,000 *g* for 15 min at 4°C. The upper aqueous phase was collected and the RNA was precipitated by adding 100% ethanol. The mixture was applied to an miRNeasy Mini spin column. After washed several times, the RNA was eluted in 26 μL of RNase-free water.

### MicroRNA microarray analysis

The discovery cohort included plasma samples from 3 patients with ALK-positive lung cancer, 3 patients with ALK-negative lung cancer, and 3 healthy volunteers. These samples were analyzed by Agilent human microRNA microarray chips (8*60 K), V21.0. The data were extract via Agilent Feature Extraction Software (v10.7). Using the quantile algorithm in GeneSpring Software 12.6 (Agilent), the raw data were normalized. MicroRNAs with differential expression of 2-fold changes or more were sifted.

### Poly(A)-tailed RNA and reverse transcription

According to methods reported previously [39], poly(A)-tailed RNAs were used in real-time reverse transcriptase polymerase chain reaction. In brief, total RNAs isolated from 100 μL of plasma, 20 μM of rATP, 1.5 μL of poly(A) polymerase reaction buffer, and 1 unit poly(A) polymerase (New England Biolabs, Hitchin, UK) were mixed in a 15-μL reaction system and incubated at 37°C for 60 min according to the manufacturer's protocol. Subsequently, poly(A)-tailed small RNA (15-μL total volume) was incubated with 2 μL of reverse-transcriptase primer (5′-GCG AGC ACA GAA TTA ATA CGA CTC ACT ATA GG (T)18(A,G or C)(A,G,C or T) -3′) at 70°C for 5 min to remove any RNA secondary structure. The reactions were chilled on ice for at least 5 min and the remaining reagents, then 8 μL of 5 × ImProm-II reverse transcription buffer, 105 μmol of MgCl2, 20 μmol of dNTP, 40 units of RNAase inhibitor, and 30 units of ImProm-II reverse transcriptase (Promega, Madison, WI, USA), were added according to the protocol. The reaction system was 40 μL, proceeding at 42°C for 30 min, 72°C for 15 min, and 20°C for 5 min.

### Identification of the stable endogenous control

Currently, there is no standard endogenous control for the circulating microRNA studies. Before the RT-qPCR, we tried to find a stable endogenous control gene for circulating microRNAs of lung cancer patients and health subjects. Thirty-four blood samples were involved, including samples from 8 patients with benign lung disease, 8 healthy subjects, 6 patients with small cell lung cancer, 6 patients with squamous cell lung cancer, and 6 patients with adenocarcinoma lung cancer. The candidate internal reference genes were chosen based on a search of the literature. Twelve candidate genes were selected, including 5s rRNA, 18s rRNA, RNU6B, RNU38B, RNU44, RNU43, RNU48, miR-221-3p, miR-93-5p, miR-191-5p, miR-103-3p, and miR-197-3p. The stable (S) value calculated from NormFinder software and the M value calculated from geNorm software were used as a screening criterion. NormFinder ranks the microRNAs by calculating stability (S) values, whereas geNorm determines the expression stabilities of the microRNAs by calculating the minimum M value. The genes with an S value below the cut-off level of 0.5 from NormFinder and with the minimum M value from GeNorm were selected as stable candidates for further RT-qPCR evaluation.

### Real-time qRT-PCR

RT-qPCR was used to measure the levels of 13 microRNAs that were differentially expressed according to microarray analysis: miR-362-5p, miR-660-5p, miR-340-5p, miR-28-5p, miR-579-5p, miR-96-5p, miR-19a-3p, miR-17-5p, miR-20a-5p, miR-24-3p, miR-107, miR-16-5p, and miR-22-3p. The primers of 5s rRNA and the 13 microRNAs for RT-qPCR are listed in Table 3. The microRNA expression level was measured using the ΔCt method, in which the Ct threshold cycle is the fractional cycle number when the fluorescence of each sample passes a fixed threshold. The relative quantification of each microRNA was performed using the chosen, stable endogenous control gene as a references gene, which was validated before. The expression levels of selected microRNAs were normalized to 5s rRNA. The results were expressed as ΔCT (Ct_miR_-Ct_ref_) for each subject. The relative expression levels of microRNAs in plasma between patients with ALK-positive NSCLC and those with ALK-negative NSCLC were calculated using the 2^−ΔΔCt^ method. The RT-qPCR was conducted on Agilent StrataGene Mx3000P Real-Time PCR System (Agilent Technologies, Germany) in 96-well plates at 95°C for 15 min, followed by 45 cycles of 94°C for 15 s, 56°C for 30 s, and then 68°C for 30 s. The specificity of polymerase chain reaction products was evaluated by the melting curve analysis.

### Statistical analysis

The data were presented as means ± standard errors of the means (SEMs) for microRNAs or as means ± standard deviations (SDs) for other variables. Relative levels of 3 microRNAs were quantified using the 2^−ΔΔCt^ method. Data were analyzed using 2-sided tests and a *p value* < 0.05 was considered statistically significant. Receiver operating characteristic (ROC) curve analyses were performed, and Youden's index was chosen to identify the optimal cut-off threshold values. The area under the ROC curve (AUC) was calculated to evaluate the specificity and sensitivity of predictions of ALK-positive NSCLC for each microRNA, as well as for the combination of microRNAs. PFS was measured from the start of crizotinib administration until the date of progressive disease, according to the RECIST. PFS was analyzed using the Kaplan-Meier method, and differences in survival were evaluated using the Logrank test. Multivariate regression analysis was conducted using the Cox proportional hazards model. These measures of diagnostic performance were estimated using GraphPad Prism 5.0 (GraphPad, La Jolla, CA, USA) and IBM SPSS 19.0 (IBM, Armonk, NY, USA) software. The selection of optimal reference genes was conducted using geNorm and NormFinder (Aarhus University Hospital, Aarhus, Denmark), as previously described [40, 41]. The Wilcoxon matched pairs signed rank sum test was used to analyze the changes of plasma levels before and after crizotinib treatment.

## Abbreviations

ALK: anaplastic lymphoma kinase
AUC: area under the curve
HS: healthy subjects
NPV: negative predictive value
NSCLC: non-small cell lung cancer
PPV: positive predictive value
ROC: receiver operating characteristic curve
RT-qPCR: real-time reverse transcriptase quantitative polymerase chain reaction
SD: standard deviation
SEM: standard error of the mean.
